# Source Apportionment and Health Risk Assessment of Potentially Toxic Elements in Shallow Groundwater Using an Integrated PMF-SOM Approach: A Case Study from Southern Dongting Lake, China

**DOI:** 10.3390/toxics14060473

**Published:** 2026-05-27

**Authors:** Xinping Deng, Bozhi Ren, Shun Zhang, Luyuan Chen, Zhaoqi Cai

**Affiliations:** 1 School of Resource & Environment and Safety Engineering, Hunan University of Science and Technology, Xiangtan 411201, China; 2 School of Earth Science and Space Information Engineering, Hunan University of Science and Technology, Xiangtan 411201, China; 3 Engineering and Mine Geological Survey and Monitor Institute of Hunan Province, Changsha 410004, China

**Keywords:** shallow groundwater around the lake, source apportionment, PMF-SOM model, health risk assessment, potentially toxic elements, water resources protection

## Abstract

Shallow groundwater in the Dongting Lake area is an important resource for domestic, agricultural, and industrial use, and its quality is essential for regional sustainable development and public health. Therefore, effective protection of this resource is urgently needed. In this paper, we integrate Positive Matrix Factorization (PMF) and Self-Organizing Map (SOM) machine-learning algorithms to conduct an in-depth analysis of the distribution, sources, and risks of toxic elements in shallow groundwater along the southern shore of Dongting Lake. The results indicate that Fe and Mn in the groundwater of the study area are at a severe pollution level, while As is at a light pollution level. The model analysis identified four pollution sources: natural sources (Fe, Mn) accounting for 31.33%, agricultural production (Zn) for 18.96%, traffic-mining mixed source (Pb, Cu, Cd) for 32.67%, and mineral dissolution-redox driven (As) for 17.04%. The average concentrations of Fe and Mn exceeded the standard limits. Although the carcinogenic metal Cd did not pose a health risk, the health risk value of As exceeded the maximum acceptable level, which requires serious attention. The PMF model quantified four potential sources of toxic elements, while SOM was used as a complementary nonlinear clustering tool to examine the consistency of the PMF-derived source contribution patterns. The integrated PMF–SOM framework, together with spatial distribution and geochemical evidence, improved the interpretability and robustness of source identification.

## 1. Introduction

Groundwater systems in lake-plain regions are vulnerable to quality degradation. Appropriate exploitation of groundwater can provide water for our life and production, but irrational exploitation will cause a series of environmental issues [1]. However, with the acceleration of industrialization, agricultural intensification, and urbanization, the shallow groundwater in the region faces the threat of potentially toxic elements pollution. Potentially toxic elements, such as As, Cd, and Pb, are not only difficult to degrade in the environment but also enriched through the food chain, causing serious harm to human health. Groundwater is one of the most important natural resources on earth [2]. And determining the origin of potentially toxic elements pollution is a prerequisite for the mitigation and management of potentially toxic elements pollution in groundwater [3].

Previous studies have extensively investigated the sources of potentially toxic elements in groundwater and their associated health risks [4]. In China, metal mining, smelting, and processing have contributed to economic development, but these activities may also release potentially toxic elements into groundwater and increase environmental and health risks. In assessing the groundwater in typical mining areas in Hunan Province, principal component analysis (PCA) of the sources of potentially toxic elements in the groundwater was used, which indicated that only by cutting off the sources could the further deterioration of groundwater pollution in mining areas be prevented [4]. Endogenous sources of toxic elements in the environment were analyzed in a typical high geologic context [5], and source contributions in the environment were quantified through spatial analysis, Absolute Principal Component Scores (APCS), and other methods [6]. A previous study investigated potentially toxic element contamination in soils around an antimony smelter in Hunan Province by integrating Geographic Information System (GIS), Positive Matrix Factorization (PMF), and health risk assessment models, and further identified the potential sources of pollution [7]. In pollution characteristic identification, many studies have found that industrial emissions and agricultural activities are the main sources of potentially toxic elements in groundwater. In addition, studies have found the key role of geological background on the natural enrichment of As, Fe, and Mn [8,9]. However, without considering the local geological environment, pollution control factors, and source apportionment, elevated concentrations of geogenic elements such as Fe and Mn may be misjudged as resulting from anthropogenic pollution. And in terms of methodology, the results of single-model analysis have a certain degree of uncertainty, and the joint analysis of multiple models has not yet been perfected.

With regard to the health risk assessment of potentially toxic elements in groundwater, the potential risks to human health from excess potentially toxic elements in groundwater include carcinogenic and non-carcinogenic risks [10]. Potentially toxic elements that pose health risks, such as Pb, Cd, and As, require special attention. Among these, Pb, as a neurotoxic element, poses a particularly significant threat to susceptible populations like children and pregnant women. Even low-level exposure can lead to severe health consequences. It is therefore a major public health concern [11,12]. Excessive levels of cancer-causing, potentially toxic elements in groundwater pose a risk to human health, and research has been conducted abroad on the contamination of groundwater with the potentially toxic element As [13]. It describes the current status of As contamination in groundwater around the globe, with more than 90 per cent of As contamination being inferred to be from ground sources. Long-term consumption of groundwater with excessive levels of As can lead to serious health problems. Thus, it is necessary to carry out a health risk assessment of potentially toxic elements in groundwater.

To address these challenges, this study focuses on shallow groundwater in the Yiyang area on the southern shore of Dongting Lake. Eight potentially toxic elements, including As, Cd, Pb, Fe, and Mn, were investigated to assess their pollution patterns, sources, and health risks. The objectives were to: (1) determine the concentration levels and spatial distribution of potentially toxic elements in shallow groundwater and evaluate pollution status; (2) integrate positive matrix factorization (PMF), self-organizing maps (SOM), spatial analysis, and hierarchical clustering to identify and quantify pollution sources more reliably; and (3) assess potential carcinogenic and non-carcinogenic health risks using a human health risk assessment model. This study provides a representative case and transferable framework for understanding potentially toxic elements sources and health risks in shallow groundwater systems of lake plains and agricultural–industrial transition zones worldwide.

## 2. Materials and Methods

### 2.1. Research Area

**Figure 1:**
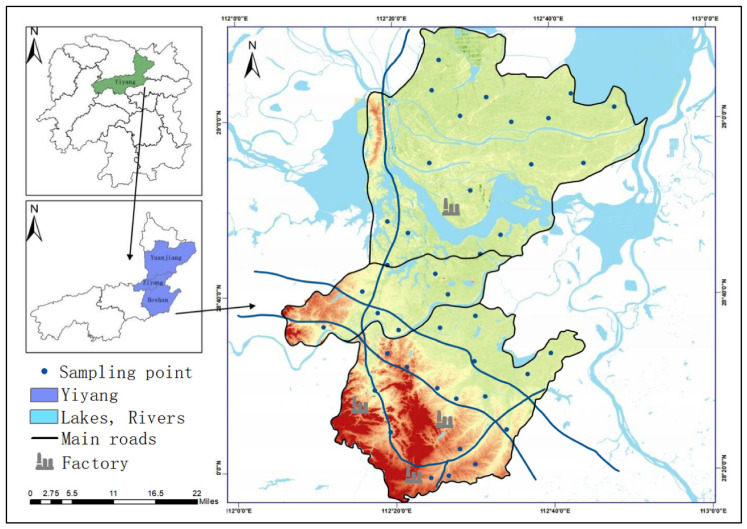
Location of the Study Area and Spatial Distribution of Sampling Points.

This study focuses on the Yiyang area on the south shore of Dongting Lake, and the specific location of the study area is shown in Figure 1. The study area, which covers Heshan District, Yuanjiang City, and Ziyang District, has geographic coordinates ranging from 27°58′38″ to 29°31′42″ N latitude and 110°43′02″ to 112°55′48″ E longitude. The terrain of the region is dominated by the Dongting Lake alluvial plain, which is flat and has a dense river network, and transitions to the hilly terrain of the remnants of the Xuefeng Mountains in the southwest. The climate belongs to a subtropical continental monsoon humid climate, with the average annual temperature of 16.1~16.9 °C, annual rainfall of 1230~1700 mm, and rainy and flood-prone in July.

The study area exhibits diverse land-use types, primarily including agricultural land such as paddy fields and drylands, urban and rural residential areas, industrial land, forest land, and water bodies. Although a separate land-use map was not included, the sampling sites were arranged to cover the main land-use settings in the study area. Specifically, sampling points in the central plain area were mainly associated with agricultural land, including paddy fields, drylands, and aquaculture-related areas; those in the southern hilly area were closer to industrial and mining activities as well as major transportation routes; and several points in the peripheral areas were located near residential, forest, and water-body settings. This sampling design allowed the study to compare groundwater potentially toxic elements characteristics under different land-use influences.

Groundwater is mainly recharged by infiltration of atmospheric precipitation. In the alluvial plain areas (the central part of the study area), groundwater flow is slow, with gentle hydraulic gradients, and discharge occurs mainly through evaporation, discharge into surface water bodies, and artificial extraction. Agricultural production extensively uses pesticides and fertilizers, while some industrial enterprises are distributed in the area, such as nonferrous metal mining and smelting, capacitor and electronic component manufacturing, and food processing. These activities may impact the quality of shallow groundwater. Heshan is primarily industrial, Yuanjiang focuses on agriculture and tourism, and Ziyang emphasizes manufacturing and mining. Based on groundwater occurrence conditions and hydraulic characteristics, groundwater in the study area can be classified into four types: loose rock pore water, red bed pore-fissure water, bedrock fissure water, and carbonate rock fissure-karst water. The study area is located in the mid-lower basin of the Zijiang River Basin, which is dominated by plains and flat terrain. Previous hydrogeochemical studies have suggested that reducing conditions may commonly occur in low-gradient alluvial plain aquifers around Dongting Lake, which can promote the reductive dissolution of Fe/Mn oxides and influence the mobility of Fe, Mn, and As [14,15]. In this study, redox conditions are discussed as a regional hydrogeochemical background.

### 2.2. Sample Collection and Testing

A total of 41 sampling points were set up in the Dongting Lake Basin and its south shore, covering different land-use types, including farmland, industrial land, and residential areas. The groundwater samples were collected from the phreatic aquifer. The depths of the sampling boreholes were approximately 15 ± 2 m below ground surface. Groundwater sampling was conducted in accordance with the relevant requirements of the Technical Specification for Environmental Monitoring of Groundwater HJ 164-2020 [16] and HJ 1019-2019 [17]. During the sampling campaign, continuous groundwater-level monitoring was not conducted; therefore, seasonal fluctuations in groundwater level were not quantitatively evaluated in this study. However, groundwater-level dynamics in the study area are closely related to seasonal precipitation, with peak groundwater levels generally lagging behind peak rainfall.

In order to ensure the data quality, the field blank samples and transportation blank samples were collected synchronously during the sampling process. After the sample is collected, protective agents (such as an acid protective agent or complexing agent) are added immediately to prevent potentially toxic elements precipitation or change, and sent to the laboratory for analysis. The potentially toxic element Fe in the water samples was measured by an ICAP 7000 SERIES (Thermo Fisher Scientific, Waltham, MA, USA) inductively coupled plasma emission spectrometer, and the detection limit of Fe was 10 μg/L. Mn, Cu, Zn, As, Cd and Pb were measured by Thermo X2 inductively coupled plasma mass spectrometer, and the detection limits of Mn, Cu, Zn, As, Cd and Pb were 0.12 μg/ L, 0.08 μg/L, 0.67 μg/L, 0.12 μg/L, 0.05 μg/L and 0.09 μg/L, respectively; Hg was measured by XGY-1011A atomic fluorescence spectrophotometer (Institute of Geophysical and Geochemical Exploration (IGGE), Langfang, China), and the detection limit of Hg was 0.04 μg/L, which ensured the accuracy and reliability of the analytical results [18,19]. The ICP-MS analysis was performed using external calibration with multi-element standard solutions. Laboratory blanks, field blanks, transport blanks, and duplicate samples were included for quality assurance and quality control. Blank values were below the corresponding method detection limits.

### 2.3. Data Analysis and Evaluation Methods

#### 2.3.1. Index Evaluation Method

The Nemerow index method is specifically divided into a single-factor pollution index and a comprehensive pollution index, and the formula for the single-factor pollution index is as follows [20]:

(1) $$P_{i}=\frac{C_{i}}{S_{i}}$$

where P_i_ is the single-factor pollution index of potentially toxic elements, $C_{i}$ is the measured value of potentially toxic elements, $S_{i}$ and is the environmental quality standard value, which is based on the Class III threshold values specified in the Standard for Groundwater Quality of China (GB/T 14848-2017 [21]).

The Nemerow index method is an evaluation method for concentration exceedance based on environmental standard values. Its classification results reflect the degree of concentration exceedance relative to the standard values, but it cannot distinguish between natural or anthropogenic causes of exceedance. The comprehensive pollution index formula is as follows [22]:

(2) $$P_{N}=\sqrt{\frac{{\bar{P}}^{2}+{P_{max}}^{2}}{2}}$$

where $P_{N}$ is the composite pollution index and $P_{max}$ is the maximum value of the single-factor pollution index. $\bar{P}$ is the average value of the single-factor pollution index of potentially toxic elements. According to the potentially toxic elements index evaluation criteria, the evaluation can be divided into four pollution degrees [23]. $P_{i}$ ≤ 1, $P_{N}$ ≤ 1; 1 < $P_{i}$ ≤ 2, 1 < $P_{N}$ ≤ 2.5; 2 < $P_{i}$ ≤ 3, 2.5 < $P_{N}$ ≤ 7; $P_{i}$ > 3, $P_{N}$ > 7, The four contamination tiers are defined as: no pollution (clean), mild pollution, moderate pollution, and severe pollution.

#### 2.3.2. Pearson Correlation Analysis and Hierarchical Cluster Analysis (HCA)

Pearson correlation analysis was performed in SPSS (version 27, IBM Corp., Armonk, NY, USA) to assess linear relationships among the eight potentially toxic elements (As, Cu, Pb, Zn, Fe, Cd, Hg, and Mn). The correlation matrix was used to identify co-occurrence patterns and potential similarities in sources or geochemical behavior [24].

Hierarchical cluster analysis (HCA) was conducted in Origin 2022 after standardizing the concentration data. HCA was applied to the elements, rather than the sampling sites, to classify them based on similarity. The dendrogram was then compared with the correlation matrix, spatial patterns, SOM results, and PMF source profiles [25,26].

#### 2.3.3. Construction of PMF-SOM Machine-Learning Models

PMF (Positive Matrix Factorization) model, the Positive Definite Matrix Factorization model, is an advanced receptor model based on factor analysis, widely used for the quantitative analysis of pollution sources in environmental media. The core of this model is the decomposition of an observation data matrix X (n × m), consisting of n samples (rows) and m chemical species (columns), into the product of two non-negative matrices: the factor contribution matrix G (n × p) and the factor composition profile matrix F (p × m), which includes a residual matrix E (n × m). Its basic mathematical model can be expressed as follows [27,28]:

(3) X=GF+E

The model’s objective is to seek the optimal solutions for G and F by minimizing the objective function Q through iterative calculations. The objective function Q is defined as the sum of squared weighted residuals:

(4) $$Q=\sum_{i=1}^{n}\sum_{j=1}^{m}(\frac{e_{ij}}{u_{ij}}{)}^{2}=\sum_{i=1}^{n}\sum_{j=1}^{m}(\frac{x_{ij}-\sum_{k=1}^{p}g_{ik}f_{kj}}{u_{ij}}{)}^{2}$$

The calculation of PMF model uncertainty data will directly affect the calculation weights of mass concentration as well as component concentration data in the PMF model, and the uncertainty is calculated according to the following formula [29]:

(5) $$U_{ij}=\sqrt{{\left(\partial \times c\right)}^{2}+{\left(0.5\times MDL\right)}^{2}}\left(c>MDL\right)$$

(6) $$U_{ij}=\frac{5}{6}\times MDL\left(c\leq MDL\right)$$

where $U_{ij}$ is the uncertainty, ∂ is the relative standard deviation, and MDL is the method detection limit.

SOM, functioning as a neural network algorithm, was initially created by the academic Kohonen [30]. The structure of the SOM network is a hexagonal or rectangular pattern, which can be divided into two different layers: one for input and the other for competition [31]. This study utilized the SOM toolbox in MATLAB R2024a to additionally calculate two core validity metrics: Quantization Error (QE) and Topological Error (TE). These metrics quantitatively characterize the clustering accuracy and the preservation of topological structure in the SOM model, clarifying the reliability of its clustering results. During SOM training, an input vector is randomly selected, and the Euclidean distance between the input vector and each neuron weight vector is calculated. The neuron with the minimum distance is identified as the best-matching unit, and the weights of this neuron and its neighboring neurons are then updated iteratively [32]:

(7) $$D_{I}=\sqrt{\sum_{i=1}^{n}\left(x_{i}(t)-w_{ij}(t\right){)}^{2}}=\left\|X-W_{j}\right\|$$

where x_i_ is the input vector of the neuron, and w_ij_ is the weight assigned to the connection between neuron i in the input layer. In this study, the number of neurons m is determined by the formula $m=5\times \sqrt{a}$. In this paper, the samples are modeled, trained, and applied through the Neural Network Clustering Toolbox in MATLAB R2024a.

The PMF and SOM models were applied to the same groundwater heavy-metal concentration dataset; however, they served different purposes. PMF was used for quantitative source apportionment by decomposing the concentration matrix into factor profiles and factor contributions. In contrast, SOM was directly applied to the standardized concentration matrix of the eight potentially toxic elements to identify nonlinear clustering patterns among sampling points and to visualize element-specific feature planes. SOM was used as a pattern recognition tool to support and validate the source interpretations obtained through PMF, correlation analysis, hierarchical clustering, and spatial distribution analysis.

#### 2.3.4. Health Risk Analysis

In this study, we examine the human health risk assessment framework introduced by the U.S. Environmental Protection Agency (USEPA) [33], which can be used for health risk evaluation of multiple environmental media and multiple sources of contamination. Health risk evaluation can link human health and groundwater contamination. In this study, the USEPA health risk assessment model was used to assess the risk of potentially toxic elements to human health in the groundwater of the study area, which consisted of potentially toxic elements that do not cause cancer (Mn, Zn, Fe, Cu, Pb, and Hg) and carcinogenic elements (As and Cd) [34]. The pathways of potentially toxic elements affecting the human body include two main aspects: the dermal infiltration pathway and the drinking water pathway [35]. The equations for the drinking water pathway (${ADD}_{i}$) and the dermal route (${ADD}_{d}$) are as follows:

(8) $$\begin{array}{c} {ADD}_{i}=\frac{C_{W}\times IR\times EF\times ED}{BW\times AT} \end{array}$$

(9) $${ADD}_{d}=\frac{C_{W}\times SA\times PC\times ET\times EF\times ED\times CF}{BW\times AT}$$

where ADD is the mean daily exposure to contaminants, C_w_ is the concentration of contaminants in water, μg/L; IR is the intake of drinking water, L/d, 2.0 for adults and 1.14 for children [36]; EF is the frequency of exposure, d/a, 350 in this study; ED is the duration of exposure, a; AT is the average time of exposure, d; For carcinogenic risk, lifetime exposure was assumed, with ED = 70 years and AT = 25,550 days. For non-carcinogenic risk, ED = 35 years and AT = 12,775 days. BW is the body weight, kg, 60 for adults and 23 for children [37]; SA is the total dermal contact surface area of the groundwater, cm^2^, 16,000 for adults and 7000 for children; PC is the dermal permeability constant of the groundwater; ET is the frequency of exposure, h/d; CF is the volume conversion. Dermal permeability constant for each potentially toxic element; ET is exposure frequency, h/d; CF is volume conversion [38].

The formulas for the non-carcinogen risk evaluation model (HQ) and the carcinogen risk evaluation model (R) are as follows [39]:

(10) $$HQ=\frac{ADD}{RfD}\times 10^{-6}$$

(11) R=SF×ADD

where HQ is the hazard factor, dimensionless; R is the health risk value for human exposure to a pollutant, dimensionless; is the average daily reference dose of the pollutant under the drinking water exposure pathway, mg/(kg·d); SF is the carcinogenicity intensity factor, kg·d/mg.

The values of RfD and SF for the eight potentially toxic elements in this study are shown in Table 1 [40].

### 2.4. Data Processing

**Figure 2:**
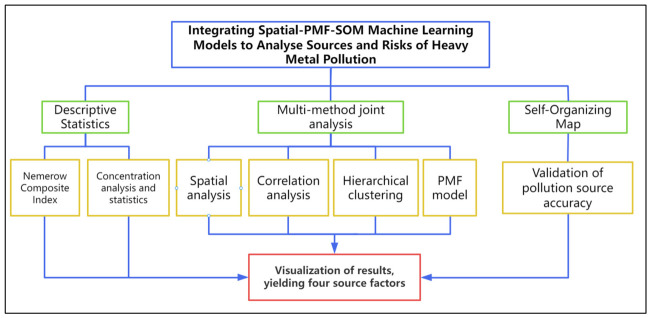
Flow chart of potentially toxic elements pollution source analysis.

In this study, Microsoft Excel was used for data processing and preliminary analysis. SPSS software (version 27, IBM Corp., Armonk, NY, USA) was employed for descriptive statistics and correlation analysis of potentially toxic elements, and ArcMap (version 10.8, Esri Inc., Redlands, CA, USA) was used to create spatial distribution maps for the eight elements. To identify contamination sources and categorize potentially toxic elements, we integrated correlation analysis, hierarchical clustering, spatial analysis, and the PMF model. The results of the clustering and the contributions of PMF factors were visualized. The SOM model was additionally used as an independent nonlinear pattern-recognition tool to examine whether the PMF-derived factor interpretations were consistent with sample clustering, element co-occurrence patterns, and spatial distribution characteristics. Hierarchical clustering was performed using Origin 2022. Source analysis was conducted using the Positive Matrix Factorization (PMF) model via EPA-PMF 5.0 software; water quality concentration and uncertainty data were imported and preprocessed for the PMF analysis. The specific workflow is shown in Figure 2.

## 3. Results and Discussion

### 3.1. Characterization of Potentially Toxic Element Concentrations in Shallow Groundwater

**Figure 3:**
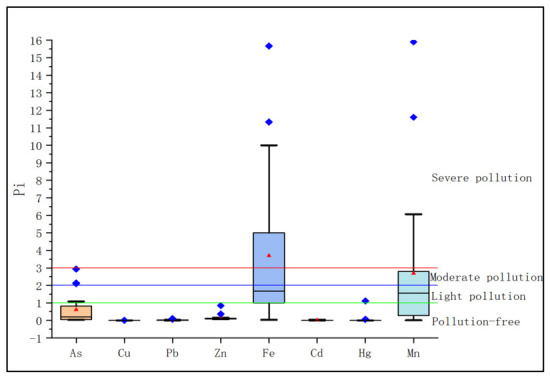
Box plot of single-factor pollution index.

Based on the calculation of the single-factor pollution index (Pi) for potentially toxic elements in the study area, it can be observed that the average Pi values for elements such as Cu, Pb, Zn, and Cd are all less than 1 (Figure 3), indicating a pollution-free status. The average Pi values for the eight potentially toxic elements are as follows: As (0.6120), Cu (0.0022), Pb (0.0221), Zn (0.1459), Fe (3.6952), Cd (0.0116), Hg (0.0614), and Mn (2.6878). Among these elements, the average Pi value of Fe was greater than 3, indicating that the average Fe concentration exceeded the Class III threshold value specified in the Standard for Groundwater Quality of China (GB/T 14848-2017) by more than three times. The average Pi value for Mn exceeds 2. Based on the calculation of the comprehensive pollution index (PN) for potentially toxic elements, except for As, Fe, and Mn, whose PN values are greater than 1, the PN values of the remaining elements are all less than 1, indicating a pollution-free status. Specifically, the PN value for As is 2.1165, classified as slight exceedance; the PN values for Fe (11.3820) and Mn (11.4025) far exceed 7, classified as severe exceedance. From the perspective of exceedance proportion across sampling points, Cu, Pb, Zn, and Cd show no exceedance at all points (accounting for 100%). The exceedance levels for Fe and Mn are more prominent, with the proportion of points where their concentrations exceed the standard limit by more than threefold being 42.86% and 19.05%, respectively. As a carcinogenic factor, As shows moderate exceedance at 14.29% of points, slight exceedance at 4.76% of points, and no exceedance at the remaining 80.95% of points, indicating that its potential risk warrants attention. For elements with high geological background values, such as Fe and Mn, it is necessary to conduct a comprehensive judgment combined with source apportionment results. Spatially, elevated Fe and Mn concentrations were mainly observed in the Dongting Lake alluvial plain. However, no statistical comparison among different groundwater types was conducted. Thus, this conclusion is based on the spatial correspondence between Fe/Mn enrichment and the alluvial-plain groundwater system, rather than on a quantitative comparison of groundwater types. The concentration and exceedance points of As are also relatively concentrated here. For types such as bedrock fissure water and carbonate fracture-karst water distributed in hilly and mountainous areas, the groundwater runoff conditions are relatively better, and the environment is more oxidizing, which is unfavorable for the reductive enrichment of Fe and Mn.

As shown in Table 2, the average concentrations were in the following order: Mn > Zn > Fe > As > Cu > Pb > Hg > Cd. The main potentially toxic elements exceeding the standards were Fe and Mn, which had high contents and showed significant discrepancies compared to the background values. Additionally, the maximum concentration of As was 29.3 μg/L, exceeding the Class III threshold value of GB/T 14848-2017. The average values of Mn and Fe also exceeded the Class III threshold value of GB/T 14848-2017 [45]. The coefficients of variation of the potentially toxic element concentrations in groundwater exceeded 1 for all except Cd [46]. The larger the coefficient, the greater the change in concentration, with the largest coefficient of 3.93 for Hg.

### 3.2. Characteristics of the Spatial Distribution of Potentially Toxic Elements in Shallow Groundwater

**Figure 4:**
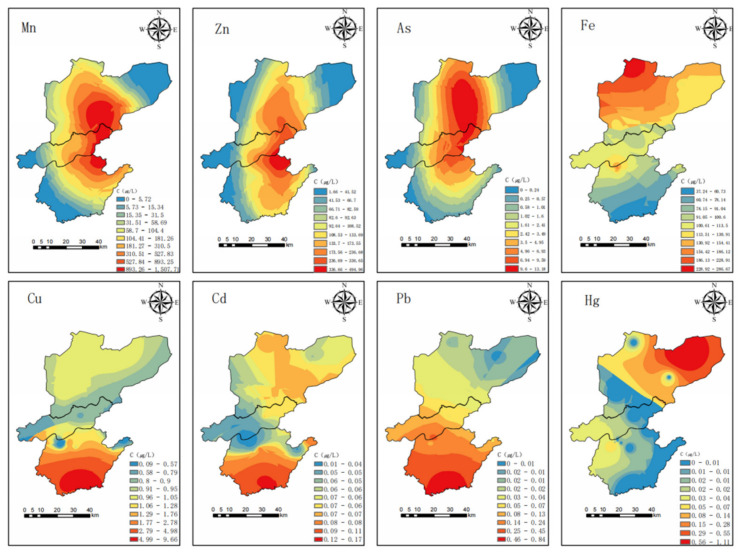
Spatial distribution of potentially toxic element concentrations in groundwater.

The spatial distribution of eight potentially toxic element concentrations in groundwater was mapped using the Inverse Distance Weighting (IDW) method in ArcGIS 10.8. Figure 4, generated from concentration data and geographic coordinates, reveals distinct spatial patterns. The concentrations of Mn and Zn exhibit similar distributions, a trend also observed for Cu, Cd, and Pb. Furthermore, hierarchical clustering categorized the metals into three distinct groups: Mn-Zn, Cu-Cd-Pb, and Hg, which is consistent with both the SOM model and the spatial distribution characteristics. High concentrations of Mn, Zn, and As are predominantly clustered in the central study area, particularly around Dongting Lake. This spatial pattern can be partly attributed to the natural geological background, as previous investigations indicate that the upstream plains of Dongting Lake have elevated natural background values for elements like Fe and Mn, exceeding regional standards [47]. This spatial pattern may be partly related to the local geological background, because previous studies have reported relatively high natural background values of Fe and Mn in the Dongting Lake plain. During prolonged hydrogeochemical processes, especially in the reducing environments of confined groundwater, Fe and Mn oxides in sediments are dissolved and released. These elements then gradually leach into the water through rainwater infiltration or groundwater percolation, leading to elevated concentrations. The enrichment of Mn, Zn, and As in the central plain shows a high degree of overlap with the Dongting Lake alluvial plain (area of porous water distribution in loose rock formations). This region features low and flat terrain, slow groundwater flow, and is prone to forming an oxygen-deficient reducing environment. Additionally, the thick lacustrine clay layers in the unsaturated zone and shallow aquifers of this area are rich in organic matter, which further promotes the reduction process and may slow down groundwater renewal, leading to the accumulation of elements. The relatively higher concentrations of Cu, Cd, and Pb in the southern part of the study area may be associated with the combined influence of industrial/mining activities and transportation routes. However, because no source-specific tracers were available, this association should be interpreted as spatial evidence rather than direct source confirmation. The average concentrations of Pb and Cd do not exceed the Class I threshold value of GB/T 14848-2017, and the Cu concentration in most samples also remains below the Class I threshold value of GB/T 14848-2017, indicating a low level of potentially toxic element contamination for Cu, Cd, and Pb.

Only one sampling point recorded a Hg concentration exceeding the standard, located in the northeastern part of the study area. The maximum Hg concentration was 1.11 μg/L, exceeding the Class III threshold value of GB/T 14848-2017 (1 μg/L). Among the remaining 40 samples, 20 had concentrations below the detection limit (0.04 μg/L), while the other 20 were below the Class I threshold value of GB/T 14848-2017 (0.1 μg/L).

### 3.3. Correlation Analysis and Hierarchical Clustering

**Figure 5:**
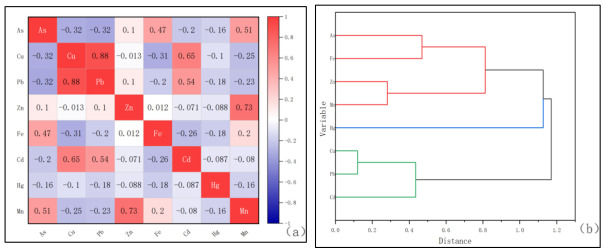
(**a**) Correlation coefficient matrix of groundwater potentially toxic elements. (**b**) Hierarchical clustering analysis results of potentially toxic elements.

To evaluate inter-element relationships, identify potential sources, and elucidate the enrichment mechanisms of potentially toxic elements, a correlation analysis was conducted. A correlation matrix for the eight potentially toxic elements (As, Cu, Pb, Zn, Fe, Cd, Hg, and Mn) was constructed using the Pearson correlation coefficient method (Figure 5a). The results reveal distinct patterns: Hg shows a low correlation with all other elements, indicating a unique contamination source. This is corroborated by the fact that only one monitoring site had a Hg concentration exceeding the Class III threshold value of GB/T 14848-2017, while others were below detection limits or complied with Class I standards. Strong positive correlations (correlation coefficients > 0.6) were observed between Pb-Cu, Mn-Zn, and Cd-Cu, suggesting shared pollution sources (such as industrial discharges or agricultural runoff) or similar geochemical behaviors [48]. Moderate correlations (coefficients between 0.45 and 0.55) for Fe-As, Mn-As, and Cd-Pb indicate potential similarities in their sources or transport pathways [49].

In order to further analyze the correlations among the distributions of potentially toxic elements, hierarchical cluster analysis (HCA) was applied to the eight potentially toxic elements. HCA is a method that hierarchically clusters samples or variables based on their similarity. In this study, HCA was performed using Origin 2022. As shown in Figure 5b, the clustering results for the eight potentially toxic elements (As, Fe, Zn, Mn, Hg, Cu, Pb, Cd) can be categorized into three main groups. The horizontal axis represents the distance between elements; a smaller distance indicates a closer relationship and higher similarity, which may imply a common pollution source. The vertical axis lists the potentially toxic elements. The HCA results suggest that the pollution sources can be classified into three clusters: Cd-Pb-Cu group: At a distance of about 0.1, Cd and Pb form the first cluster. Between distances of 0.4 and 0.5, Cu joins to form a cluster with Cd and Pb, indicating that Cd may share similar pollution sources with Pb and Cu. Around industrial land and major transportation routes, Cd, Pb, and Cu consistently form tightly clustered groups, which verifies the source identification of potentially toxic elements.

Mn-Zn-Fe-As group: At a distance of 0.2–0.3, Mn and Zn cluster together, suggesting possible common sources such as agricultural fertilization, mining activities, or natural mineral weathering. At around distance 0.8, Fe joins the Mn–Zn cluster, implying that these three elements may originate from similar natural or geological backgrounds. Between distances 0.4 and 0.5, As clusters with the Mn–Zn–Fe group, indicating similarity in sources, which may include agricultural practices and mining emissions. Independent Hg group: Hg clusters with the other elements only at a relatively large distance, indicating that its distribution characteristics are distinct from those of the other potentially toxic elements, and that its pollution sources are relatively independent. In the porous water of loose rock formations in the Dongting Lake alluvial plain area, the reductive hydrogeochemical environment is prominent. The clustering relationship among Fe, Mn, and As is extremely close (linkage distance < 0.5), indicating a natural geochemical process where the dissolution of iron-manganese oxides jointly controls As release under reducing conditions. In regions dominated by agricultural land (paddy fields, dry farmland), Mn and Zn often form a tight cluster, which confirms that agricultural activities (such as fertilizer and pesticide application) serve as an important common input pathway for these elements.

### 3.4. PMF-SOM Machine-Learning Source Parsing Results

To accurately identify the potential sources of eight potentially toxic elements and provide data support for groundwater management and ecological restoration in the area, this study applied the Positive Matrix Factorization (PMF) model. Based on the PMF results, four potential contamination sources were identified, and their respective contributions were quantified. However, due to the inherent characteristics of the PMF model, the results inevitably contain a degree of uncertainty. To address this limitation and validate the PMF findings more comprehensively, the Self-Organizing Map (SOM) model was introduced. The combination of PMF (which provides quantitative source apportionment) and SOM (which captures nonlinear features in the data) significantly improves the accuracy and reliability of pollution source identification. Specifically, the SOM model visualizes sampling points as neuron matrices (Figure 7) and integrates spatial distribution information from GIS, correlation analysis, and hierarchical clustering results to verify the pollution sources inferred by the PMF model.

To evaluate the robustness of the model and determine the optimal number of factors, we systematically tested solutions with three to six factors. The results showed that the three-factor model led to mixed source profiles, making it difficult to clearly distinguish between geological and anthropogenic sources, and its environmental significance was ambiguous. Although the five-factor and six-factor models slightly improved the fit for Cd and Mn, they introduced factors with very low contribution rates and ambiguous physical meaning, posing a significant risk of overfitting. The finally selected four-factor model had an objective function value (Q) closest to the expected value (Qexp) calculated based on the degrees of freedom (Q/Qexp ≈ 1) [50,51]. Therefore, although the R^2^ values for Cd and Mn under the four-factor solution are relatively low, they represent the optimal solution for the current dataset. However, through multiple verifications, including spatial analysis, SOM clustering, and geochemical mechanisms, its source apportionment conclusions are generally reliable.

**Figure 6:**
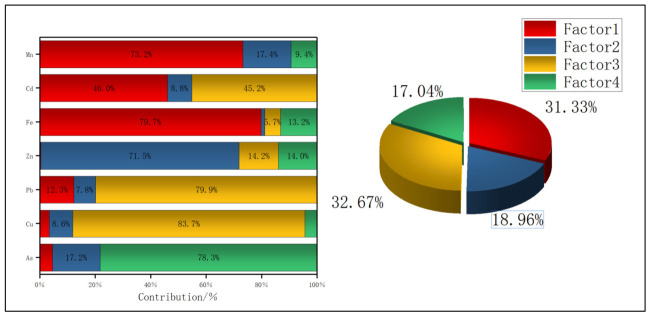
Source analysis of potentially toxic elements in groundwater based on PMF modeling.

**Figure 7:**
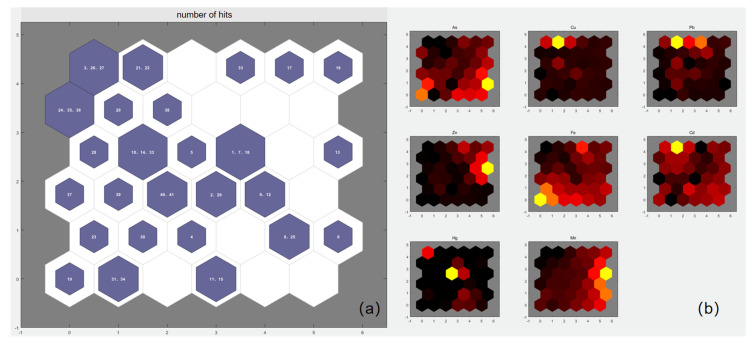
SOM results based on the standardized heavy-metal concentration matrix: (**a**) distribution of groundwater sampling points on the SOM; (**b**) element-specific SOM feature planes for heavy-metal concentrations.

The pollution sources of potentially toxic elements and the corresponding contribution ratio of the factors are shown in Table 3 and Figure 6. In Figure 7a, each hexagonal neuron represents a unit in the Self-Organizing Map, where the placement of sampling points reflects the similarity of their overall elemental concentrations. Closer neurons indicate samples with more similar profiles. Figure 7b shows the feature planes for individual elements, in which the color intensity of each neuron indicates the average standardized concentration of that element within the neuron. Brighter colors (e.g., yellow) correspond to higher concentrations, while darker colors (e.g., black) indicate lower concentrations. This visualization allows identification of spatial patterns and co-occurrence of elements, complementing the PMF source apportionment. After the SOM model completed the clustering of the standardized concentration data from the 41 sampling points, the calculated Quantization Error was QE = 0.14, and the Topological Error was TE = 0.11. These results indicate that the model possesses high clustering accuracy and good preservation of the topological structure.

The main contributing elements of Factor 1 are Fe (79.7%), Cd (46.0%), and Mn (73.2%). The study area belongs to the Dongting Lake region, and some studies have shown that Fe and Mn contamination of the groundwater in the Dongting Lake is generally due to the factors of the geological environment, and that the geological structure and soil properties of the Dongting Lake region determine a high natural background value of the Fe and Mn elements in the groundwater. The groundwater system is a typical plain-type groundwater system, and the environment of groundwater as a whole is relatively reduced, which has an important influence on the morphology and content of Fe and Mn [52]. Furthermore, related research has revealed that the core control of Fe-Mn cycling in shallow groundwater lies in hydrogeochemical redox processes [15]. Both PMF and SOM point to the main source of Factor 1 as the natural geological background, and the results of both corroborate each other, which strengthens the reliability of the conclusions. However, the potential influence of well casing materials on Fe and Mn concentrations was not quantitatively assessed in this study and should be considered in future work.

The main contributing element of Factor 2 was Zn (71.5%), followed by As (17.2%) and Mn (17.4%). As can be seen from Figure 4, the central part of the study area with a high concentration of elemental Zn is mostly farmland as well as ponds, where feed, pesticides, and chemical fertilizers used in fisheries and agricultural activities may contain elemental Zn [53]. These chemicals enter the water body through surface runoff and infiltration, further exacerbating Zn pollution, and their spatial distribution highly overlaps with the highlighted area of the SOM (near point 13). The spatial overlap between elevated Zn concentrations and agricultural/aquaculture-related areas suggests that Factor 2 may be influenced by agricultural inputs, including fertilizers, pesticides, and feed residues. Therefore, Factor 2 was interpreted as an agricultural-related input factor. However, because specific agricultural tracers were not measured, this interpretation should be regarded as a plausible source hypothesis rather than direct confirmation.

Factor 3 is primarily contributed by Cu (83.7%) and Pb (79.9%), followed by Cd (45.2%). The spatial distribution of these three elements is also highly similar, with their high-value zones concentrated in the southern part of the study area (Figure 4). Cu, Pb, and Cd are densely distributed in the upper-left highlighted area of the SOM matrix, matching the spatial gradient distribution of the southern mining area. This region contains sites of historical mining activities and is adjacent to major transportation routes. It should be emphasized that, due to the high similarity in the elemental profiles of Cu, Pb, and Cd between traffic emissions and mining activities, and the lack of specific tracers (such as isotope ratios or characteristic organic indicators) in this study, the PMF model is currently unable to strictly distinguish their independent contributions. Therefore, Factor 3 should be understood as a mixed factor with distinct anthropogenic source characteristics, rather than a precise quantification of the respective contributions of traffic and mining. Notably, the strong correlation between Cu and Pb (r = 0.88, *p* < 0.01) supports their common source, while the involvement of Cd may indicate associated pollution from mining waste, such as Cd-bearing ores. Cu, Pb, and Cd are typical composite pollution marker elements. Traffic emissions (brake pad wear, tire wear, historical leaded gasoline residues) and nonferrous metal mining/smelting activities can all release these elements; in receptor models, it is difficult to completely separate them [54,55]. Car tires contain metallic elements such as Pb and Cu, and dust from tire wear is dispersed in the wind and enters the water body indirectly [56]. Some studies have shown that Pb is a signature element in traffic emissions, mainly from the combustion of leaded petrol, vehicle exhaust emissions, etc. Car tire and brake pad wear, fuel and lubricant leakage, etc., contain Cu and Pb [57]. After the sampling points were mapped by the SOM model, a sample cluster characterized by the Cu-Pb-Cd contribution combination was clearly identified (highlighted area on the upper left). This clustering result, from the perspective of sample similarity, verifies that Factor 3 parsed by PMF—which exhibits high homology for the Cu-Pb-Cd element group—objectively exists, and its spatial distribution is highly correlated with the southern industrial/mining activity area. Similar findings have also been reported in other aquatic environments. For example, Machado da Silva Acioly et al. investigated potentially toxic and essential elements in sediments and found that metal accumulation patterns were closely associated with multiple anthropogenic pressures. This suggests that the Cu–Pb–Cd-associated factor in shallow groundwater may reflect the combined influence of multiple human activities rather than a single isolated source [58]. It reveals that Factor 3 is primarily attributed to Traffic-mining mixed source.

Factor 4 is primarily characterized by its contribution to As (78.3%). According to the concentration distribution map (Figure 4), areas exceeding the concentration standard are all located in the central part of the study area. Furthermore, the correlation coefficients between As-Mn and As-Fe are 0.51 and 0.47, respectively, indicating a strong positive correlation. Studies suggest that the main source of As is significantly influenced by geological origins. As originates from As-bearing minerals within the geological formations of the study area, and its migration is likely controlled by redox conditions. In reducing groundwater environments, the dissolution of As-bearing minerals (such as pyrite) and the reductive dissolution of Fe/Mn oxides may jointly promote the release of As. Microbial metabolism of organic matter creates reducing conditions, which drive the reductive dissolution of iron oxides. Under reducing hydrogeochemical conditions, the reductive dissolution of Fe/Mn oxides may promote the release of adsorbed or co-precipitated As into groundwater. Since dissolved organic carbon and microbial indicators were not measured in this study, organic-matter-driven microbial reduction is discussed only as a possible mechanism and is not used as direct evidence for source interpretation [59]. Factor 4 is thus inferred to represent mineral dissolution. The overlapping distribution of As and Fe in the SOM (dark area in the upper left corner), combined with the PMF result for Factor 4 (78.3% contribution from As), reveals that Factor 4 corresponds to mineral dissolution. Therefore, Factor 4 is interpreted as “Mineral Dissolution—Redox Driven.”

In this study, through the in-depth synergy of Positive Matrix Factorization (PMF) model and Self-Organized Mapping (SOM), combined with Geographic Information System (GIS) spatial analysis and hierarchical clustering algorithms, we accurately quantified the correlation degree between potentially toxic elements indicators and analyzed the spatial distribution characteristics of potentially toxic elements in depth. A multi-dimensional pollution source analysis system was constructed to comprehensively and systematically identify and analyze the pollution sources of potentially toxic elements through the coupled analysis and in-depth integration of multiple methods. The four source factors are natural sources, agricultural production, traffic-mining mixed source, and mineral dissolution—redox driven.

### 3.5. Health Risk Assessment

**Figure 8:**
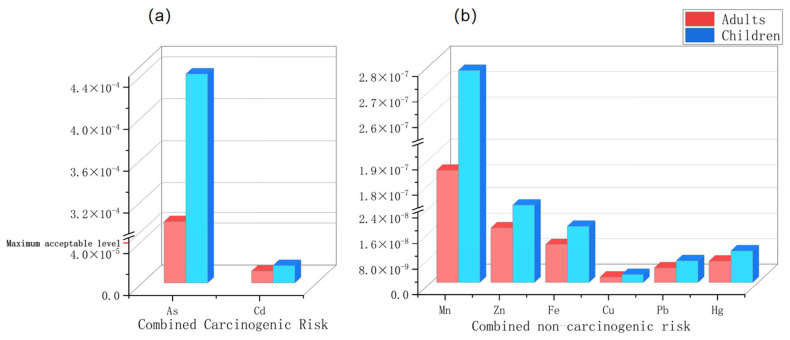
(**a**) Histogram of combined carcinogenic health risks. (**b**) Histogram of combined non-carcinogenic health risks.

The results of the health risk calculations for the drinking water route and the dermal route are shown in Table 4. The maximum acceptable level recommended by ICRP is 5.00 × 10^−5^ [34]. According to the USEPA guidelines, carcinogenic effects are based on lifetime exposure and therefore do not distinguish between age groups. It can be seen from Figure 8 that the per capita combined risk of the six non-carcinogenic potentially toxic elements did not exceed the maximum acceptable level, but the health risk value of As reached 3.00 × 10^−4^, exceeding the maximum acceptable level of 5.00 × 10^−5^. The maximum concentration of potentially toxic elements in As was 29.30 μg/L, exceeding the Class III threshold value of GB/T 14848-2017 [60]. The health risk value of Cd in carcinogenic factors did not exceed the maximum acceptable level of 5.00 × 10^−5^, the maximum concentration of Cd was 0.22 μg/L, the maximum value did not exceed the national groundwater level II standard, and the health risk caused by Cd was small. Asbestos is one of the primary factors causing carcinogenic health risks [61]. Moreover, from the spatial distribution and the analysis of pollution sources, it can be seen that, especially in the groundwater of the central Dongting Lake region, the health risk value of As is relatively high and needs to be emphasized. In this study area, As was identified as a key factor leading to lifelong carcinogenic risk. This result is consistent with the reports of several global high As areas, such as Bangladesh and Argentina, indicating that the public health threat caused by natural hydrogeochemical processes is a common problem across borders. In addition to focusing on its carcinogenic risk, greater vigilance should be exercised against the chronic health hazards posed by long-term consumption, such as damage to the nervous and cardiovascular systems [62,63]. In agricultural irrigation, As accumulates within the food chain; therefore, it is recommended that future assessments incorporate multi-pathway exposure analysis. Furthermore, existing risk assessment models contain uncertainties when extrapolating from high-dose to low-level environmental exposures, and their estimates should not be regarded as precise results [64]. Consequently, when making environmental management decisions based on these findings, a safety margin should be allowed, and intervention measures should be prioritized.

The health risk values of the six non-carcinogenic potentially toxic elements ranged from 2.29 × 10^−13^ to 2.78 × 10^−7^, which did not exceed the maximum acceptable level. Among them, the non-carcinogenic health risk value of Cu was the smallest, and the comprehensive risk values of adults and children were 1.67 × 10^−9^ and 2.47 × 10^−9^, respectively. The non-carcinogenic health risk value of Mn was the largest, and the comprehensive risk values of adults and children were 1.88 × 10^−7^ and 2.78 × 10^−7^. According to the health risk assessment criteria, the six non-carcinogenic potentially toxic elements had little health risk to the human body, and the daily activities of the population would not cause obvious harm. However, the average concentration of potentially toxic elements in Fe and Mn exceeded the Class III threshold value of GB/T 14848-2017. According to the spatial distribution of potentially toxic element concentration, the concentration of potentially toxic elements in Fe and Mn exceeded the standard in the central and northern parts of the study area. In the use of underground drinking water, the underground drinking water with Fe and Mn exceeding the standard can be treated by physical, chemical, and biological methods, which need to be paid attention to.

**Table 4:** Per capita health risks from the drinking water route and the dermal infiltration route.

Event	Element	Drinking Water Route		Skin Penetration Route		Combined Risk	
Carcinogenic	As	2.93 × 10 ^−4^		6.53 × 10 ^−6^		3.00 × 10 ^−4^	
	Cd	1.13 × 10 ^−5^		5.74 × 10 ^−8^		1.14 × 10 ^−5^	
Total Health Risk		3.05 × 10 ^−4^		6.59 × 10 ^−6^		3.11 × 10 ^−4^	
		Adults	Children	Adults	Children	Adults	Children
Non-Carcinogenic	Mn	1.87 × 10 ^−7^	2.78E × 10 ^−7^	7.50 × 10 ^−10^	5.64 × 10 ^−10^	1.88 × 10 ^−7^	2.78 × 10 ^−7^
	Zn	1.55 × 10 ^−8^	2.31 × 10 ^−8^	1.42 × 10 ^−9^	1.06 × 10 ^−9^	1.70 × 10 ^−8^	2.42 × 10 ^−8^
	Fe	1.18 × 10 ^−8^	1.75 × 10 ^−8^	3.98 × 10 ^−11^	2.99 × 10 ^−11^	1.18 × 10 ^−8^	1.75 × 10 ^−8^
	Cu	1.65 × 10 ^−9^	2.46 × 10 ^−9^	1.67 × 10 ^−11^	1.26 × 10 ^−11^	1.67 × 10 ^−9^	2.47 × 10 ^−9^
	Pb	4.51 × 10 ^−9^	6.71 × 10 ^−9^	3.05 × 10 ^−13^	2.29 × 10 ^−13^	4.51 × 10 ^−9^	6.71 × 10 ^−9^
	Hg	6.54 × 10 ^−9^	9.73 × 10 ^−9^	5.97 × 10 ^−11^	4.48 × 10 ^−11^	6.60 × 10 ^−9^	9.77 × 10 ^−9^
Total Health Risk		2.27 × 10 ^−7^	3.37 × 10 ^−7^	2.28 × 10 ^−9^	1.72 × 10 ^−9^	2.29 × 10 ^−7^	3.39 × 10 ^−7^

### 3.6. Policy and Management Suggestions

Based on the source apportionment results of the PMF-SOM model (natural sources 31.33%, agricultural activities 18.96%, traffic-mining mixed source 32.67%, mineral dissolution 17.04%) and the health risk assessment, this study proposes the establishment of a management system characterized by “zonal classification and targeted measures.” For the southern hilly area, management should focus on traffic-related pollution and mining activities, including stricter control of mining, smelting, tailings storage, industrial wastewater discharge, and road runoff. For the central agricultural plain, priority should be given to reducing Zn-containing agricultural inputs, optimizing fertilizer and pesticide use, improving aquaculture feed management, and establishing ecological buffer zones. For the Dongting Lake alluvial plain, where Fe, Mn, and As are more likely linked to geogenic background and redox processes, efforts should focus on drinking water safety, screening of contaminated wells, Fe/Mn/As removal facilities, and alternative water supplies.

We also added more specific suggestions for future work, such as increasing sampling density in different hydrogeological units, sampling in both wet and dry seasons, measuring in situ parameters including Eh, DO, pH, EC, and DOC, and incorporating high-resolution land-use and well-construction data. These revisions make the management implications and future research directions more closely tailored to the study area.

## 4. Conclusions

In this study, we systematically analyzed the characteristics and risks of toxic elements in shallow groundwater along the south shore of Dongting Lake, China, by integrating the PMF-SOM machine-learning method. The results are as follows:

(1) The order of average concentrations of potentially toxic elements in the shallow groundwater of this region is: Mn > Zn > Fe > As > Cu > Pb > Hg > Cd. Among them, the average concentrations of Fe and Mn significantly exceed the Class III groundwater quality standards, indicating severe concentration exceedance; the average concentration of As is at a slight exceedance level; the concentrations of the remaining metals all remain within the standard limits. Except for Cd, the coefficients of variation for the concentrations of the other potentially toxic elements are all greater than 1, indicating strong spatial heterogeneity in their distribution.

(2) Mn, Zn, and As are mainly distributed in the middle of the study area, and the elemental concentrations of Cu, Cd, and Pb are mainly distributed in the south of the study area, with a small degree of potentially toxic element contamination of Cu, Cd, and Pb. Hg elemental concentration has only one point with exceeded values, located in the northeast of the study area, where Hg exceeds the standard. This area needs to be monitored intensively to determine the contamination situation.

(3) The PMF model estimated four potential source-related factors, and the SOM, spatial distribution, correlation analysis, and hierarchical clustering results provided complementary evidence for interpreting these factors. Revealing four primary pollution sources: natural origins (Fe, Mn), agricultural activities (Zn), combined traffic-mining mixed source (Pb, Cu, Cd), and Mineral Dissolution-Redox Driven (As). Among these, the combined source of traffic-mining mixed source was the most significant contributor (32.67%), followed by natural origins (31.33%), agricultural activities (18.96%), and mineral dissolution (17.04%). These findings underscore that effectively controlling industrial, agricultural, and traffic emissions is a priority for protecting local groundwater resources.

(4) The health risk assessment showed that the health risk levels of the six non-carcinogenic metals Fe, Mn, Cu, Zn, Hg, and Pb did not exceed the maximum acceptable level and posed a relatively small health risk to humans. However, the average concentration of Fe and Mn exceeded the standard and still required attention. The range of health risk values for the carcinogenic metal As was 4.90 × 10^−6^ to 4.36 × 10^−4^, which exceeded the maximum acceptable level. Therefore, the potential for toxic As pollution needs to be taken seriously. Especially in the central part of the study area, such pollution directly threatens the safety of drinking water, highlighting the necessity of emergency and long-term treatment measures to protect public health in key areas of As pollution.

## Figures and Tables

**Table 1:** Values of parameters relevant for health risk evaluation.

Element		PC (1 × 10^−3^)	SF(kg·d/mg)		RfD (mg/(kg·d))		Source
			Drinking Water Route	Dermal Route	Drinking Water Route	Dermal Route	
Carcinogenic	As	1.8	1.5	3.66	-	-	USEPA IRIS (https://www.epa.gov/iris, accessed on 21 May 2026)
	Cd	1	6.1	6.1	-	-	USEPA IRIS (https://www.epa.gov/iris, accessed on 21 May 2026)
Non-Carcinogenic	Mn	0.1	-	-	0.046	0.0018	[41]
	Zn	0.6	-	-	0.3	0.01	[41]
	Fe	0.1	-	-	0.3	0.045	[42]
	Cu	0.6	-	-	0.04	0.012	[43]
	Pb	0.004	-	-	0.0014	0.00042	[44]
	Hg	1.8	-	-	0.0003	0.0003	[44]

**Table 2:** Groundwater potentially toxic elements’ concentration analysis statistics (μg/L).

Elemental	As	Cu	Pb	Zn	Fe	Cd	Hg	Mn
Average value (μg/L)	6.12	2.07	0.20	145.90	**110.48**	0.06	0.06	**268.78**
Minimum value (μg/L)	0.27	ND	ND	69.10	ND	ND	ND	0.92
Maximum value (μg/L)	**29.30**	10.10	1.02	843.00	**470.00**	0.22	1.11	**1590.00**
Standard deviation (μg/L)	8.26	3.25	0.32	171.02	130.90	0.05	0.24	403.50
Coefficient of variation	1.35	1.57	1.61	1.17	1.18	0.94	3.93	1.50
Variance	68.19	10.55	0.10	29,247.97	17,133.66	0.00	0.06	162,810.57
Kurtosis	2.36	2.73	1.53	15.32	1.59	2.58	20.61	5.99
Skewness	1.75	2.02	1.66	3.82	1.48	1.13	4.52	2.45
Class III threshold value of GB/T 14848-2017	10	1000	10	1000	30	5	1	100
Class I threshold value of GB/T 14848-2017	1	10	5	50	10	0.1	0.1	50

Note: Class I and Class III threshold values are based on the Standard for Groundwater Quality of China (GB/T 14848-2017). ND indicates not detected. Bold values indicate concentrations exceeding the Class III threshold values of GB/T 14848-2017.

**Table 3:** PMF resolution results (%).

Elemental	Factor 1	Factor 2	Factor 3	Factor 4
As	4.5	17.2	0.0	78.3
Cu	3.3	8.6	83.7	4.4
Pb	12.3	7.8	79.9	0.0
Zn	0.3	71.5	14.2	14.0
Fe	79.7	1.4	5.7	13.2
Cd	46.0	8.8	45.2	0.0
Mn	73.2	17.4	0.0	9.4

## Data Availability

The data presented in this study are available from the corresponding author upon reasonable request.
